# Putative Astroglial Dysfunction in Schizophrenia: A Meta-Analysis of ^1^H-MRS Studies of Medial Prefrontal Myo-Inositol

**DOI:** 10.3389/fpsyt.2018.00438

**Published:** 2018-09-21

**Authors:** Tushar Kanti Das, Avyarthana Dey, Priyadharshini Sabesan, Alborz Javadzadeh, Jean Théberge, Joaquim Radua, Lena Palaniyappan

**Affiliations:** ^1^Department of Psychiatry, University of Western Ontario, London, ON, Canada; ^2^Robarts Research Institute, London, ON, Canada; ^3^Lawson Health Research Institute, London, ON, Canada; ^4^Department of Medical Biophysics, University of Western Ontario, London, ON, Canada; ^5^FIDMAG Germanes Hospitalàries, CIBERSAM, Sant Boi de Llobregat & Institut d'Investigacions Biomediques August Pi i Sunyer (IDIBAPS), Barcelona, Spain

**Keywords:** myo-inositol, astroglia, schizophrenia, inflammation, spectroscopy

## Abstract

**Background:** Several lines of evidence support a role for astroglial pathology in schizophrenia. Myo-inositol is particularly abundant in astroglia. Many small sized studies have reported on myo-inositol concentration in schizophrenia, but to date these have not been pooled to estimate a collective effect size.

**Methods:** We reviewed all proton magnetic resonance spectroscopy (1H-MRS) studies reporting myo-inositol values for patients satisfying DSM or ICD based criteria for schizophrenia in comparison to a healthy controls group in the medial prefrontal cortex published until February 2018. A random-effects model was used to calculate the pooled effect size using *metafor* package. A meta-regression analysis of moderator variables was also undertaken.

**Results:** The literature search identified 19 studies published with a total sample size of 585 controls, 561 patients with schizophrenia. Patients with schizophrenia had significantly reduced medial prefrontal myo-inositol compared to controls (RFX standardized mean difference = 0.19, 95% CI [0.05–0.32], *z* = 2.72, *p* = 0.0067; heterogeneity *p* = 0.09). Studies with more female patients reported more notable schizophrenia-related reduction in myo-inositol (*z* = 2.53, *p* = 0.011).

**Discussion:** We report a small, but significant reduction in myo-inositol concentration in the medial prefrontal cortex in schizophrenia. The size of the reported effect indicates that the biological pathways affecting the astroglia are likely to operate only in a subset of patients with schizophrenia. MRS myo-inositol could be a useful tool to stratify and investigate such patients.

## Introduction

A role for astroglial pathology has been long suspected in schizophrenia (1–3). Astrocytes are critical for reducing oxidative stress and restoring redox balance in the brain, thus preventing neurotoxicity (4, 5). Astrocytes enable the crucial glutamate-glutamine cycle that helps clear extracellular glutamate from synaptic space as well as reduce the deleterious cellular ammonia content (6, 7). In addition, two crucial indicators of neuronal connectivity—synaptic maintenance and myelination—appear to rely on astrocytic guidance (8–10). Thus, abnormalities in astrocytic function can produce neuronal dysconnectivity as well as glutamatergic abnormalities that are known to occur in schizophrenia (11). Indeed, converging genetic and molecular evidence now supports the case for a primary role of astroglial dysfunction in schizophrenia (10, 12).

*In vivo* imaging of astrocytic integrity holds promise in clarifying the nature of its dysfunction in schizophrenia. ^1^H-MRS does not specifically differentiate between brain cell types; nevertheless, given that myo-inositol is particularly abundant in astroglia rather than the neurons and other cells, it can be considered an astroglial marker (13, 14). The MRS measure of myo-inositol predominantly reflects astrocytic intracellular compartment, where it has osmotic functions (15, 16). An increase in MRS myo-inositol resonance relates to markers of astroglial activation (17, 18), associated with gliosis (19, 20), and occurs in response to brain injury (21, 22), thus reflecting an inflammatory response. On the other hand, myo-inositol also has an important role as an intracellular second messenger in calcium mediated glutamatergic signaling (23). Reduced myo-inositol resonance may relate to astroglial dysfunction and consequently, aberrant extracellular glutamate clearance from synaptic space. Thus, low levels of myo-inositol may in turn facilitate excitotoxic damage and local inflammatory processes that are currently subjects of investigation in the pathophysiology of schizophrenia (1).

Many small sized studies have reported on myo-inositol concentration in schizophrenia, but to date these have not been pooled to estimate a collective effect size (24). Examining the state of myo-inositol abnormalities will aid in our understanding of the role of astroglial cells in schizophrenia. We reviewed MRS studies reporting myo-inositol resonance in schizophrenia and conducted a meta-analysis to synthesize the nature of myo-inositol abnormalities in the medial prefrontal cortex of patients with schizophrenia. We focussed on the medial prefrontal cortex as most MRS studies in schizophrenia have placed voxels in this brain region (24).

## Methods

### Search process

We followed the guidelines set out by the consensus statement from PRISMA group (25). Our literature search started with the MEDLINE electronic database to identify journal articles published until 28 February 2018. We used the following Medical Subject Headings and freeform search terms: (schizophrenia OR schizo^*^ OR psychos^*^ OR psychot^*^) AND (“1H-MRS” OR “1H NMRS” OR “1HMRS” OR “MRS” OR “Magnetic resonance spectroscopy” OR “Spectroscopy” OR “proton magnetic resonance spectroscopy”) AND (“myo-inositol” OR “inositol” OR “myo-inositol”). We noted that in many reports, myo-inositol was reported as a secondary measure, and not included in keywords or abstracts. As a result, we used the terms (“glutathione” OR “NAA” OR “n-acetyl aspartate” OR “glutamine” OR “GSH” OR “neurometabolic” OR “Glutamate” OR “Glu” OR “GABA” OR “Lactate” OR “creatinine”) instead of the 3 terms denoting myo-inositol in order to identify all eligible studies. We attempted to contact authors whenever the individual studies indicated that myo-inositol resonance of adequate quality was measured in the brain region of interest (see below) but when the data was not published. We also undertook a manual search of reference lists of review articles and eligible full text articles. Third, we repeated the search with Google Scholar to identify journal articles that were not indexed on MEDLINE. Finally, we also searched the citation records of Google Scholar for all identified full text articles in order to locate in press articles that are not yet indexed. Two authors (AD and PS) undertook independent searches using the inclusion and exclusion criteria without any exchange of notes.

### Inclusion/exclusion criteria

Peer-reviewed articles in English language reporting myo-inositol concentrations in the brain in patients with schizophrenia or schizoaffective disorder in comparison with a healthy control group were included. We did not include studies that only report on patients with bipolar disorder or depression related psychosis. We selected studies where the largest proportion of MRS voxel was placed on the medial prefrontal cortex, anterior to the posterior commissure, as per the cingulate boundaries defined by Vogt et al. (26). This ensured that both caudal and rostral ACC placements were included, but posterior cingulate voxels were excluded. In line with Egerton et al. (27), we will use the term medial frontal cortex (mFC) to describe this region of distributed voxel placement.

We excluded 1H-MRS studies that reported within-subject changes in myo-inositol without the required group comparison contrast and studies that excluded adult samples of age >16. If a single study was reported as 2 samples, the largest sample was included. In case of partial overlap, both studies were included with weighting based only on the non-overlapping sample for the smaller study (28, 29). We also excluded studies where no information was available on voxel placement (30) or when study-specific Cramer-Rao Lower Bound (a measure of MRS signal quality and reliability) was exceeded for myo-inositol signal (31).

We extracted the study-specific mean and standard deviation of 1H-MRS myo-inositol concentration for the control and patient groups. As the meta-analysis was based on effect size from group differences, we included absolute as well as ratio measures of myo-inositol concentrations, as long as both patients and controls in a dataset had identical metrics reported. When a study reported on more than 1 demographically stratified patient group, all contrasts were included in the meta-analysis (29); when groups were stratified according to clinical characteristics (e.g., treatment response) but compared against a single control group, the contrasts were combined to form a single dataset (weighted mean and pooled SD for a single patient group) (32). When voxels were split into 2 hemispheres, average values were computed (mean value from the 2 hemispheres and pooled SD). We contacted authors when these values were not reported or if moderator variables for meta-regression were not available.

Meta-analysis was conducted using the *metafor* package of R CRAN (33). We used a random-effects model to calculate the pooled effect size, with 95% confidence limits. This approach enables more robust inferences when there is a notable heterogeneity among individual studies. We assessed heterogeneity using I^2^ statistics for quantification and Cochran's Q for statistical significance test. Potential publication bias was quantified using Egger's test. Sensitivity testing was carried out using a jack-knife approach. During each of the iterations of this leave-one-out jack-knife testing, one study was left out and the meta-analytical estimate for (n-1) studies was recalculated. Meta-regression analyses were undertaken to investigate the effect of (1) age (based on mean age of patients) (2) gender (based on % female patients) (3) medication status (based on % unmedicated patients) (4) scanner strength (in Tesla) and (5) duration of illness (based on mean years of illness).

## Results

### Search results

**Figure 1:**
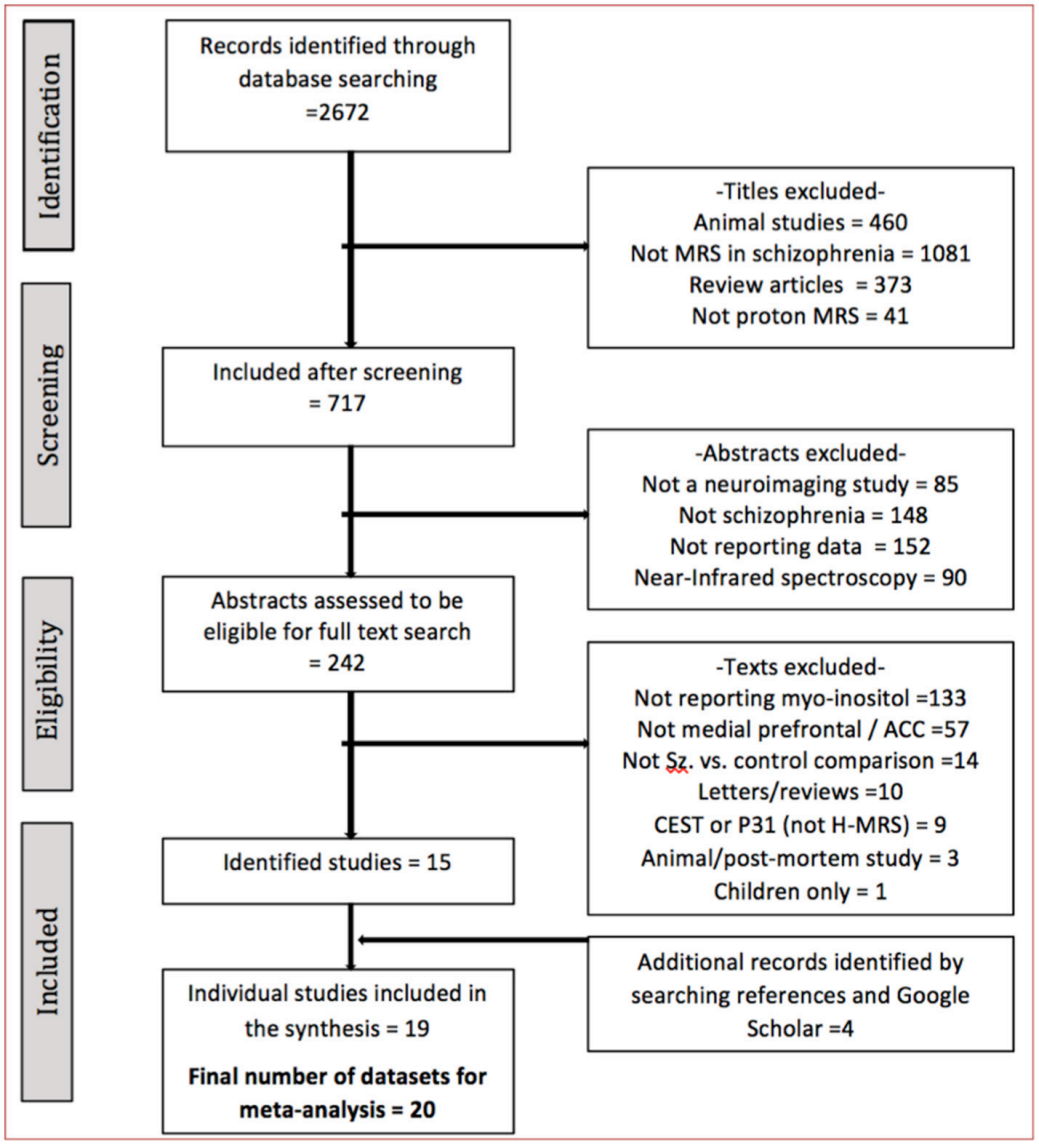
The literature search process.

The literature search identified 19 studies (one with 2 eligible contrasts (29)), published between 2002 and 2018, with a total of 561 patients and 585 controls (PRISMA flow diagram presented Figure 1) (29, 32, 34–49). The sample sizes ranged from 10 to 75 for controls and 9–72 for patients (Table 1). Mean illness duration varied between 0.49 and 27.4 years.

**Table 1:** Clinical and demographic features of studies included in the meta-analysis.

**Study**	**Year**	**Number of patients/controls**	**Patients mean mI (SD)**	**Controls Mean mI (SD)**	**% Female patients**	**Age patient Mean (SD) in years**	**Age controls Mean (SD) in years**	**Unmedicated patients in %**	**Illness duration Mean (SD) in years**
Theberge	2002	20/20	8.82 (3.92)	8.72 (2.71)	35.00	25.40 (7.20)	25.52 (7.29)	100	1.75 (2.00)
Delamillieure	2002	17/14	0.63(0.19)	0.66(0.13)	17.65	31.25(6.09)	30.14(6.39)	29.41	8.42(5.45)
Theberge	2003	21/21	9.73 (3.1)	8.74 (3.38)	4.76	37.10 (10.60)	33.30 (11.73)	0	15.6 (8.92)
Yasukawa	2005	15/20	0.98 (0.28)	1.29 (0.24)	46.67	32 (4.9)	36.1 (6.8)	13.33	1.7 (2.10)
Ongur	2008	17/21	0.92(0.33)	1.09(0.32)	41.18	41.8 (9.8)	34.3(10)	0	NA
Tayoshi	2009	30/25	6.73 (2.2)	8.22 (2.35)	53.33	33.8 (9.5)	34.9 (10.7)	0	10.3 (8.7)
Ongur	2010	21/19	0.25 (0.03)	0.27 (0.04)	33.33	39 (10.8)	36.3 (9.8)	13.33	21.1 (7.3)
Shirayama	2010	19/18	5.7 (0.68)	5.55 (0.72)	36.84	30.5 (5.6)	31.4 (8.4)	5.26	7.3 (5.2)
Lutkenhoff	2010	9/21	7.90(1.73)	8.26(2.65)	44.44	48.8(11.5)	55.7(3.8)	0	27.4(11.1)
Bustillo	2014	72/75	10.97 (5.47)	11.03 (4.96)	15.28	36.43 (14.25)	35.04 (12.17)	3.7	15.9 (5.5)
Demjaha	2014	14/10	6.88 (1.66)	6.39 (1.07)	54.17	44.8 (10.9)	44.2 (8.9)	0	16.2(9.4)
Chiappelli	2015	59/69	6.84 (0.84)	6.94 (0.60)	30.51	37.1 (10.6)	33.3 (11.73)	5.08	NA
Brandt	2016	24/24	6.36 (1.34)	6.69 (0.88)	20.83	37.5 (16.7)	36.6 (14.6)	0.00	NA
Rowland	2016a	45/53	6.8 (0.9)	6.90 (0.60)	35.56	37.7 (12.8)	37.1 (13.1)	8.9	14.7 (12.1)
Rowland	2016b	27/29	5.8 (0.64)	5.80 (0.42)	37.04	34.4 (13.1)	29.7 (9.4)	18.52	13.1 (12.1)
Chiu	2017	19/14	5.13 (2.65)	7.71 (1.63)	42.11	29.11 (6.68)	27.71 (5.88)	0	1.47 (1.23)
Taylor	2017	16/18	8.4 (1.3)	8.00 (0.80)	18.75	22.7 (2.9)	23.9 (4.6)	6.06	2.46 (1.31)
Wijtenburg (young)	2017	48/54	6.6 (0.7)	6.72 (0.50)	29.17	25.2 (4.5)	25.2 (4.8)	8.33	6.1 (5.8)
Wijtenburg (older)	2017	47/39	6.82 (0.9)	7.00 (0.80)	44.68	49.5 (5.4)	51.2 (5.7)	4.26	25.4 (9.3)
Reid	2018	21/21	4.88 (0.47)	5.03 (0.50)	23.81	23.2 (4.4)	23.5 (4.5)	4.76	0.49 (0.86)

*mI, myo-inositol (absolute or ratio measure of concentration)*.

**Figure 2:**
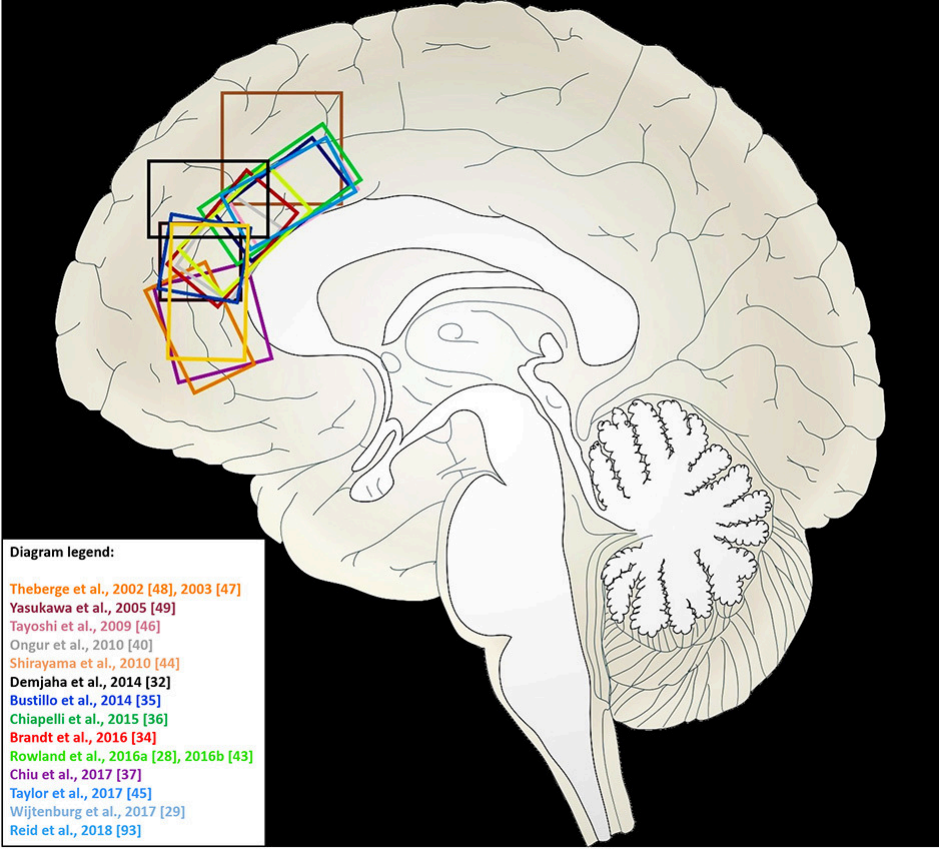
Voxel locations in medial frontal cortex for 1H-MRS studies of myo-inositol included in this meta-analysis. Studies from which a sagittal view of the MRS voxel could not be obtained are not included in this illustration.

The voxel placement of individual studies is shown in Figure 2. MRS parameters for individual studies are shown in Table 2.

**Table 2:** MRS parameters of individual studies.

**Study**	**Year**	**Field strength (T)**	**MRS fitting model**	**Voxel size/volume (cm × cm × cm) or cc**	**MRS sequences**	**TE/TR (ms)**
Theberge	2002	4	fitMAN	1.0 × 1.5 × 1.0	STEAM	20/2,000
Delamillieure	2002	1.5	In-house analysis	3 × 1.5 × 2.5	STEAM	30/1,500
Theberge	2003	4	fitMAN	1.0 × 1.5 × 1.0	STEAM	20/2,000
Yasukawa	2005	1.5	In-house analysis	1.8 cc	PRESS	30/1,500
Ongur	2008	4	LCModel	2.0 × 2.0 × 2.0	PRESS	48/2,000
Tayoshi	2009	3	LCModel	1.7 × 1.7 × 1.5	STEAM	18/5,000
Ongur	2010	4	LCModel	2.3 × 2.2 × 2.3	MEGA-PRESS	30/5,000
Shirayama	2010	3	LCModel	2.8 × 3.0 × 2.2	PRESS	68/2,000
Lutkenhoff	2010	3	LCModel	2.0 × 2.0 × 2.0	PRESS	30/3,000
Bustillo	2014	3	LCModel	2.0 × 2.0 × 3.0	PRESS	40/1,500
Demjaha	2014	3	LCModel	2.0 × 2.0 × 2.0	PRESS	30/3,000
Chiappelli	2015	3	LCModel	4.0 × 3.0 × 2.0	PR-STEAM	6.5/2,000
Brandt	2016	7	LCModel	3.0 × 2.0 × 1.2	STEAM	28/3,000
Rowland	2016a	3	LCModel, GannetFit	4.0 × 3.0 × 2.0	PR-STEAM	14/3,000
Rowland	2016b	7	LCModel	3.0 × 2.0 × 2.0	STEAM	6.5/2,000
Chiu	2017	3	GannetFit	3.0 × 3.0 × 3.0	MEGA-PRESS	68/2,000
Taylor	2017	7	fitMAN	2.0 × 2.0 × 2.0	STEAM	10/3,000
Wijtenburg (young)	2017	3	In house analysis	3.0 × 4.0 × 2.0	PR-STEAM	6.5/2,000
Wijtenburg (older)	2017	3	In house analysis	3.0 × 4.0 × 2.0	PR-STEAM	6.5/2,000
Reid	2018	7	LCModel	2.7 × 2.0 × 1.0	STEAM	5/10,000

*LCModel, Linear Combination Model; STEAM, STimulated Echo Acquisition Mode; PRESS, Point REsolved Spectroscopic Sequence; MEGA-PRESS, MEshcher-GArwood Point RESolved Spectroscopy; PR-STEAM, Phase Rotation STimulated Echo Acquisition Mode; TE/TR, Echo Time/Repetition Time*.

### Meta-analysis results

**Figure 3:**
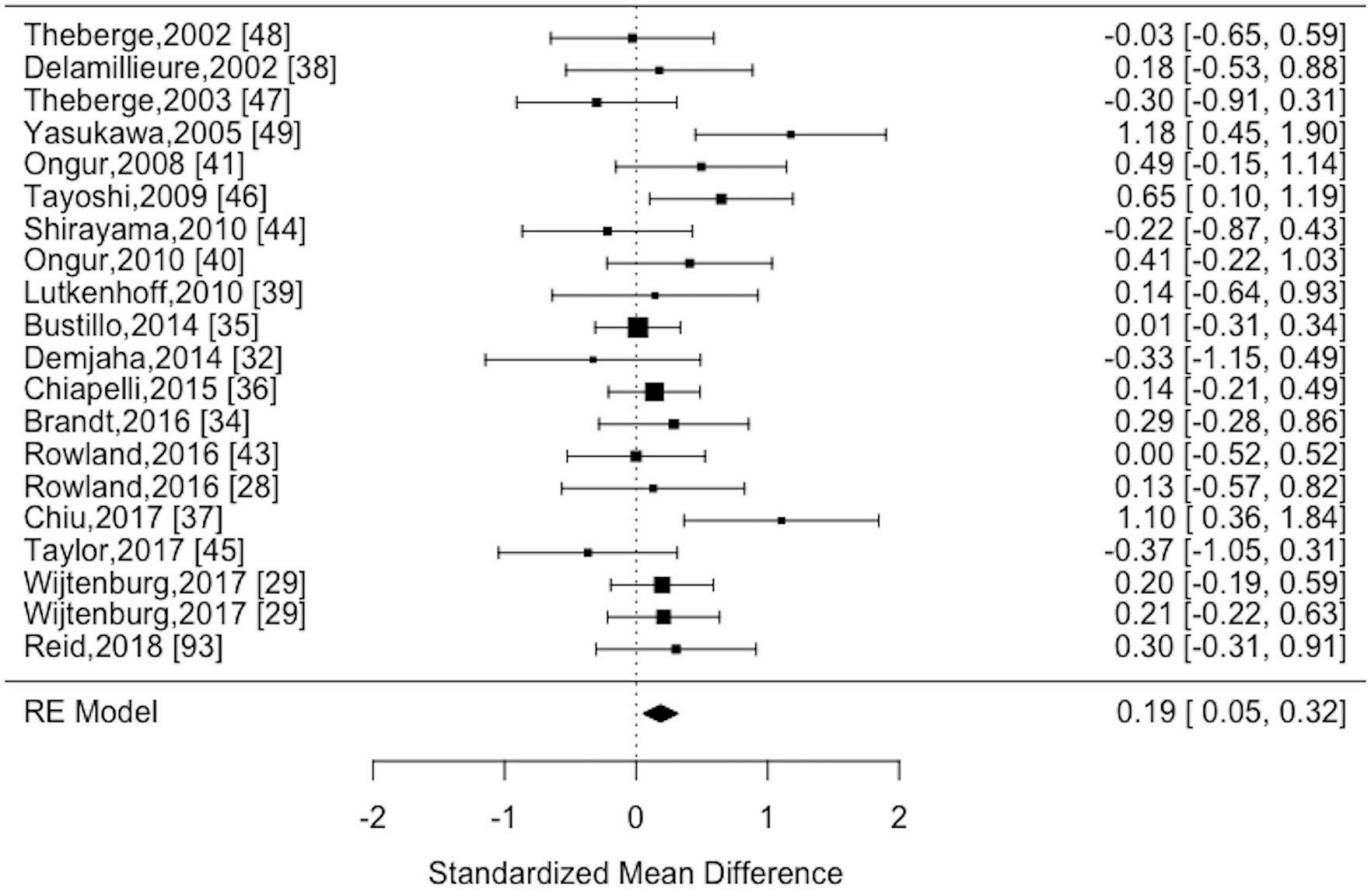
Forest plot of MRS myo-inositol studies comparing schizophrenia vs. healthy controls.

The estimate of heterogeneity had a trend level statistical significance (I^2^ = 14.61%; Cochran's Q = 27.64, *p* = 0.09) among the 20 datasets eligible for analysis. Random effects analysis revealed reduced myo-inositol content in patients with schizophrenia compared to healthy controls (effect estimate = 0.19, 95% CI [0.05–0.32], *z* = 2.72, *p* = 0.0067). These results are displayed in the forest plot Figure 3.

### Sensitivity/bias analysis

**Figure 4:**
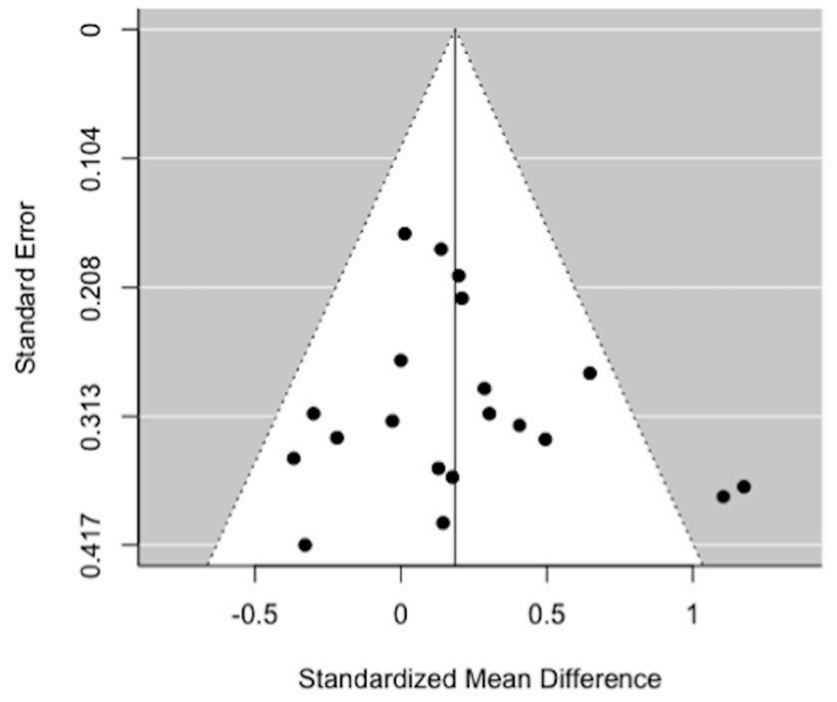
Funnel plot of MRS myo-inositol studies comparing schizophrenia vs. healthy controls.

All the 16 iterations of the leave-one out analyses were statistically significant, indicating that the meta-analytical estimates were reliable and not influenced by any single study. Egger's test for funnel plot asymmetry (Figure 4) was not statistically significant (*t* = 0.77, *p* = 0.45), indicating low probability of publication bias.

### Meta-regression analysis

**Figure 5:**
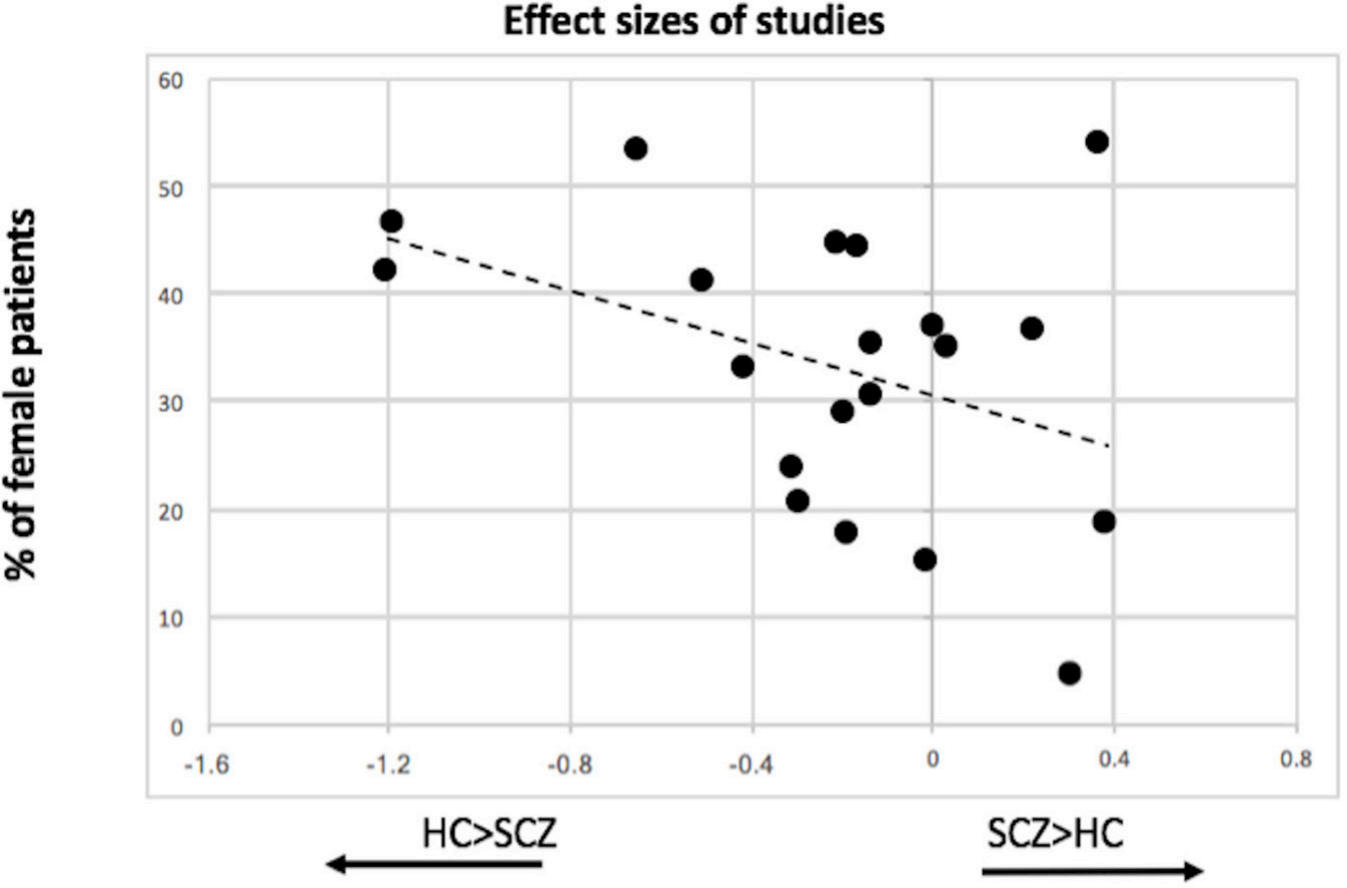
Association between proportion of female patients included in individual MRS studies and the effect size of myo-inositol resonance.

There was a statistically significant moderator effect of the percentage of female patients included in the samples in the effect size for myo-inositol (*z* = 2.53, *p* = 0.011). With this moderator, heterogeneity significantly decreased (Cochran's Q = 21.2, *p* = 0.27). Specifically, studies with more female patients were more likely to report reduced myo-inositol concentrations in patients compared to controls (Figure 5). We did not find any statistically significant moderator effect of the proportion of unmedicated patients (*z* = −0.60, *p* = 0.55), scanner strength (*z* = −1.21, *p* = 0.22), echo time (*z* = 1.59, *p* = 0.11), repetition time (*z* = 0.13, *p* = 0.9), age of patients (*z* = −0.05, *p* = 0.95), and duration of illness (*z* = −0.94, *p* = 0.34).

## Discussion

The main finding from this meta-analysis is the observation of a small, but statistically significant reduction in myo-inositol concentration in the medial frontal cortex in schizophrenia. There is a notable heterogeneity across MRS studies; a substantial proportion of this heterogeneity is explained by the sex distribution in individual studies. In studies with higher number of female patients, the myo-inositol reduction is much more pronounced. We found no evidence of publication bias, and the meta-analytic estimates were sensitive to removal of any of the individual studies. These results indicate that myo-inositol reduction in medial frontal cortex occurs in some patients with schizophrenia, especially in a subset that is more likely to include female patients.

To our knowledge, this is the first meta-analysis of MRS myo-inositol studies in schizophrenia. Post-mortem studies in schizophrenia indicate a reduction in frontal myo-inositol (50) as well as reduced glial cell count (51), of 32–35% in layer 5 (52, 53) and 20% in layer 6 of the prefrontal cortex (54), though contradicting results indicating normal (55, 56) or increased glial cell counts also exist (57, 58). In this context, reduced myo-inositol resonance reported in our meta-analysis, when taken together with reduced glutamate levels reported in established cases of schizophrenia (59), may reflect deficits in astrocyte activation and recruitment [as proposed in (21)], rather than an actual reduction in the cell count.

Our meta-regression analysis indicates that studies with female subjects are more likely to report lower myo-inositol resonance among patients. An association between sex and myo-inositol has not been reported so far in schizophrenia (36, 46). Interestingly, Chiappeli et al. reported that depressive symptoms, rather than sex, are associated with lower myo-inositol in schizophrenia (36). Both reductions in myo-inositol (60) and glial loss (61) in the medial prefrontal cortex are reported in depressive disorder. Given that one-third of patients with schizophrenia require antidepressant treatments (62), it is possible that myoinositol reduction is prominent in a subgroup of patients prone to depression. We were not able to test this notion, as except for Chiappeli et al. other MRS studies have not reported on the distribution of affective symptom severity among patients with schizophrenia. Nevertheless, it is worth noting that depression is much more common among women, than men with schizophrenia (63). Sex-specific epigenetic differences have been noted in the enzymes that regulate myo-inositol turnover in rat tissues (64). Importantly, astrocytes exhibit sexual dimorphism during development (65) and in their response to inflammation in later life (66, 67). Further investigations in larger samples of female human subjects, and in patients with and without affective symptoms are warranted.

Meta-analyses of medial prefrontal MRS studies suggest that glutamate (68), N-acetyl aspartate levels (69) are reduced in schizophrenia indicating possible dendritic reduction (70), while no consistent changes are noted in GABA concentration (27) or pH levels (71). Glutamate levels are higher during early stages of schizophrenia, but appear reduced in older cohorts with more established illness (68). We did not observe any age- or illness duration related effects on myo-inositol reduction, suggesting that astroglial dysfunction could be an invariant feature of schizophrenia, possibly contributing to the observed course of glutamatergic abnormalities. Preclinical studies suggest that at excitotoxic levels of glutamatergic signaling, inositol turnover could be notably reduced. In this context, the putative dysfunction of synaptic transmission in schizophrenia could share a common origin, simultaneously affecting the neuron-astroglia network. Similar to NAA, myo-inositol also reflects cellular membrane integrity. Thus a combined NAA and myo-inositol changes could reflect the status of dendritic spine development or loss, as shown in preclinical studies (72). While the existing MRS literature cannot be taken as conclusive due to several technical limitations (as highlighted in the meta-analyses cited above), the observations to date make a compelling case to consider astrocytic dysfunction in further detail in schizophrenia.

There are several caveats that need to be considered when interpreting the results reported here. We limited our analysis to medial prefrontal cortex, as the number of studies examining other brain regions is limited and voxel placements are more diverse. As a result, the observed myo-inositol reduction may not be generalizable to other brain regions. In fact, an increase in MRS myo-inositol signal has been reported in regions such as basal ganglia (73) and parietal lobe (74) in patients with schizophrenia, while a reduction occurs in medial temporal white matter (75). Secondly, mood stabilizers acutely deplete inositol levels (76). None of the included studies reported on the use of mood stabilizer drugs in the patient samples. The effect of antipsychotics on myo-inositol concentration is hitherto unknown. Antipsychotics can reduce astrocyte count, and thus contribute to reduced myo-inositol concentration (77), though regional differences can be expected from existing data (78). We did not find any systematic association between either unmedicated patient numbers or duration of illness (which often relates also to cumulative antipsychotic exposure in clinical settings) to the reported effect sizes. Furthermore, both schizophrenia and antipsychotics can affect metabolite relaxation rates (mostly T1, but likely also T2) (79–81). Therefore, the choice of acquisition technique, at a given field strength, could affect the ability to detect a difference between patients and controls. Nevertheless, we did not observe any linear relationship between echo time, scanner strength, repetition time and effect sizes reported in individual studies. We noted several studies where MRS sequences were suitable to extract myo-inositol concentrations alongside other metabolites, but myo-inositol levels were not measured or reported. Though our estimate of publication bias was low, it is likely that MRS myo-inositol concentration is largely underreported in the literature. Finally, we did not include analysis that primarily contrasted bipolar disorder or depression with psychosis with healthy controls or patients with schizophrenia. Thus, the observed changes in myo-inositol cannot be taken to be specific for schizophrenia.

Prenatal exposure to maternal immune activation (MIA) reduces cingulate cortex myo-inositol in mice, which in turn relates to physiological markers of schizophrenia phenotype such as deficits in pre-pulse inhibition and reduced glutamic acid decarboxylase (GAD_67_) levels (82). These changes were reversed when the offspring were exposed to a *n-3 polyunsaturated fatty acid* (PUFA) enriched post-weaning diet (82). Consistent with this observation, healthy human subjects who have reduced omega-3 fatty acid profile (measured from erythrocytes), show reduced medial prefrontal myo-inositol and exhibit slower reaction times in a continuous performance task (83). We speculate that these observations, considered alongside the reported reduction in myo-inositol levels in schizophrenia, may indicate a specific developmental perturbation. It is worth noting that dietary replacements may not have the same intended effect across disorders; for example, in major depressive disorder where myo-inositol level is reduced, inositol supplementation appears to be beneficial (84, 85), though similar effects have not been observed in schizophrenia (86, 87). Studies that investigate the effect of dietary interventions on brain myo-inositol levels in specific diagnostic subgroups are warranted to further understand the translational potential of such approaches.

It is important to note that both increased (21, 22) and reduced (88–91) brain myo-inositol levels have been noted in various inflammatory states. Thus, the reduced myo-inositol level noted in schizophrenia does not contradict the role of neuroinflammation in this illness. In fact, this observation adds an important clarification that the inflammatory changes observed to date may be secondary to a permissive astrocytic environment, whereby reduced myo-inositol levels in astrocytes facilitate osmotic damage, as well as glutamatergic excess. Without longitudinal data that tracks pre-psychotic and post-psychotic changes in same individuals, this notion of primary astrocytic dysfunction should be considered to be merely speculative.

In summary, in patients with schizophrenia, a small but statistically significant reduction in medial prefrontal myo-inositol resonance is observable. The size of the reported effect indicates that the biological pathways affecting the myo-inositol system are likely to operate only in a subset of patients with schizophrenia. In this regard, MRS myo-inositol could be a useful tool to parse heterogeneity as well as to explore treatment stratification in schizophrenia. Furthermore, combining MRS myo-inositol measurement with *in-vivo* probes of astroglial function (e.g., PET ligands selective for the astrocytic imidazoline binding sites (92)) could take this investigation further in the near future.

## Author contributions

LP conceived, designed, supervised the analysis, and wrote the draft manuscript. TD undertook the statistical analysis, prepared figures and tables, and contributed to writing the manuscript. JR undertook the statistical analysis and contributed to writing the manuscript. AD and AJ undertook literature search, prepared figures/tables, and contributed to writing the manuscript. PS undertook literature search, verified extracted data, and contributed to writing the manuscript. JT contributed to the conception of the review and contributed to writing the manuscript.

### Conflict of interest statement

LP reports personal speaker/advisory fees from Otsuka Canada, Janssen Canada, SPMM Course Limited, UK, Canadian Psychiatric Association; book royalties from Oxford University Press; investigator-initiated educational grants from Janssen Canada, Otsuka Canada outside the submitted work. The remaining authors declare that the research was conducted in the absence of any commercial or financial relationships that could be construed as a potential conflict of interest.

## References

[1] Bernstein H-G Steiner J Bogerts B. Glial cells in schizophrenia: pathophysiological significance and possible consequences for therapy. Exp Rev Neurother. (2009) 9:1059–71. 10.1586/ern.09.59 19589054

[2] Harrison PJ. Neuropathology of schizophrenia. Psychiatry (2005) 4:18–21. 10.1383/psyt.2005.4.10.187600178

[3] Schnieder TP Dwork AJ. Searching for neuropathology: gliosis in Schizophrenia. Biol Psychiatr. (2011) 69:134–9. 10.1016/j.biopsych.2010.08.027 21035789 PMC3593070

[4] Bélanger M Allaman I Magistretti PJ. Brain energy metabolism: focus on astrocyte-neuron metabolic cooperation. Cell Metab. (2011) 14:724–38. 10.1016/j.cmet.2011.08.016 22152301

[5] Dringen R Pfeiffer B Hamprecht B. Synthesis of the antioxidant glutathione in neurons: supply by astrocytes of cysgly as precursor for neuronal glutathione. J Neurosci. (1999) 19:562–9. 10.1523/JNEUROSCI.19-02-00562.1999 9880576 PMC6782200

[6] Rose CF Verkhratsky A Parpura V. Astrocyte glutamine synthetase: pivotal in health and disease. Biochem Soc Trans. (2013) 41:1518–24. 10.1042/BST20130237 24256247

[7] Schousboe A Scafidi S Bak LK Waagepetersen HS McKenna MC. Glutamate metabolism in the brain focusing on astrocytes. Adv Neurobiol. (2014) 11:13–30. 10.1007/978-3-319-08894-5_2 25236722 PMC4667713

[8] Bélanger M Magistretti PJ. The role of astroglia in neuroprotection. Dialog Clin Neurosci. (2009) 11:281–95. 19877496 10.31887/DCNS.2009.11.3/mbelanger PMC3181926

[9] Volterra A Meldolesi J. Astrocytes, from brain glue to communication elements: the revolution continues. Nat Rev Neurosci. (2005) 6:626–40. 10.1038/nrn1722 16025096

[10] Windrem MS Osipovitch M Liu Z Bates J Chandler-Militello D Zou L. Human iPSC glial mouse chimeras reveal glial contributions to schizophrenia. Cell Stem Cell (2017) 21:195–208.e6. 10.1016/j.stem.2017.06.012 28736215 PMC5576346

[11] Xia M Abazyan S Jouroukhin Y Pletnikov M. Behavioral sequelae of astrocyte dysfunction: focus on animal models of schizophrenia. Schizophrenia Res. (2016) 176:72–82. 10.1016/j.schres.2014.10.044 25468180 PMC4439390

[12] Duncan LE Holmans PA Lee PH O'Dushlaine CT Kirby AW Smoller JW. Pathway analyses implicate glial cells in schizophrenia. PLoS ONE (2014) 9:e89441. 10.1371/journal.pone.0089441 24586781 PMC3933626

[13] Glanville NT Byers DM Cook HW Spence MW Palmer FBSC. Differences in the metabolism of inositol and phosphoinositides by cultured cells of neuronal and glial origin. Biochim Biophys. Acta (1989) 1004:169–79. 10.1016/0005-2760(89)90265-8 2546591

[14] Hattingen E Raab P Franz K Zanella FE Lanfermann H Pilatus U. Myo-inositol: a marker of reactive astrogliosis in glial tumors? NMR Biomed. (2008) 21:233–41. 10.1002/nbm.1186 17562554

[15] Brand A Richter-Landsberg C Leibfritz D. Multinuclear NMR studies on the energy metabolism of glial and neuronal cells. Dev Neurosci. (1993) 15:289–98. 10.1159/000111347 7805581

[16] Griffin JL Bollard M Nicholson JK Bhakoo K. Spectral profiles of cultured neuronal and glial cells derived from HRMAS (1)H NMR spectroscopy. NMR Biomed. (2002) 15:375–84. 10.1002/nbm.792 12357551

[17] Filibian M Frasca A Maggioni D Micotti E Vezzani A Ravizza T. *In vivo* imaging of glia activation using ^1^H-magnetic resonance spectroscopy to detect putative biomarkers of tissue epileptogenicity. Epilepsia (2012) 53:1907–16. 10.1111/j.1528-1167.2012.03685.x 23030308

[18] Rothermundt M Ohrmann P Abel S Siegmund A Pedersen A Ponath G. Glial cell activation in a subgroup of patients with schizophrenia indicated by increased S100B serum concentrations and elevated myo-inositol. Prog Neuro-Psychopharmacol Biol Psychiatr. (2007) 31:361–4. 10.1016/j.pnpbp.2006.09.013 17081670

[19] Geiszler PC Ugun-Klusek A Lawler K Pardon M-C Yuchun D Bai L. Dynamic metabolic patterns tracking neurodegeneration and gliosis following 26S proteasome dysfunction in mouse forebrain neurons. Sci Rep. (2018) 8:4833. 10.1038/s41598-018-23155-2 29555943 PMC5859111

[20] Pardon M-C Yanez Lopez M Yuchun D Marjanska M Prior M Brignell C. Magnetic resonance spectroscopy discriminates the response to microglial stimulation of wild type and Alzheimer's disease models. Sci Rep. (2016) 6:19880. 10.1038/srep19880 26813748 PMC4728482

[21] Harris JL Choi I-Y Brooks WM. Probing astrocyte metabolism *in vivo*: proton magnetic resonance spectroscopy in the injured and aging brain. Front Aging Neurosci. (2015) 7:202. 10.3389/fnagi.2015.00202 26578948 PMC4623195

[22] Kierans AS Kirov II Gonen O Haemer G Nisenbaum E Babb JS. Myoinositol and glutamate complex neurometabolite abnormality after mild traumatic brain injury. Neurology (2014) 82:521–8. 10.1212/WNL.0000000000000105 24401686 PMC3937862

[23] Kim H McGrath BM Silverstone PH. A review of the possible relevance of inositol and the phosphatidylinositol second messenger system (PI-cycle) to psychiatric disorders–focus on magnetic resonance spectroscopy (MRS) studies. Hum Psychopharmacol. (2005) 20:309–26. 10.1002/hup.693 15880397

[24] Schwerk A Alves FDS Pouwels PJW van Amelsvoort T. Metabolic alterations associated with schizophrenia: a critical evaluation of proton magnetic resonance spectroscopy studies. J Neurochem. (2014) 128:1–87. 10.1111/jnc.12398 23937509

[25] Moher D Liberati A Tetzlaff J Altman DG The PRISMA Group preferred reporting items for systematic reviews and meta-analyses: the PRISMA statement. PLoS Med. (2009) 6:e1000097 10.1371/journal.pmed.1000097 19621072 PMC2707599

[26] Vogt BA Nimchinsky EA Vogt LJ Hof P. Human cingulate cortex: surface features, flat maps, and cytoarchitecture. J Compar Neurol. (2004) 359:490–506. 10.1002/cne.903590310 7499543

[27] Egerton A Modinos G Ferrera D McGuire P. Neuroimaging studies of GABA in schizophrenia: a systematic review with meta-analysis. Transl Psychiatr. (2017) 7:e1147. 10.1038/tp.2017.124 28585933 PMC5537645

[28] Rowland LM Summerfelt A Wijtenburg SA Du X Chiappelli JJ Krishna N. Frontal glutamate and γ-aminobutyric acid levels and their associations with mismatch negativity and digit sequencing task performance in schizophrenia. JAMA Psychiatr. (2016) 73:166–74. 10.1001/jamapsychiatry.2015.2680 26720179 PMC4740214

[29] Wijtenburg SA Wright SN Korenic SA Gaston FE Ndubuizu N Chiappelli J Altered glutamate and regional cerebral blood flow levels in schizophrenia: A1H-MRS and pCASL study. Neuropsychopharmacology (2017) 42:562–71. 10.1038/npp.2016.172 27562377 PMC5399238

[30] Delamillieure P Fernandez J Constans J-M Brazo P Benali K Abadie P. Proton magnetic resonance spectroscopy of the medial prefrontal cortex in patients with deficit schizophrenia: preliminary report. Am J Psychaitry (2000) 157:641–3. 10.1176/appi.ajp.157.4.641 10739430

[31] Théberge J Williamson KE Aoyama N Drost DJ Manchanda R Malla AK. Longitudinal grey-matter and glutamatergic losses in first-episode schizophrenia. Br J Psychiatry (2007) 191:325–34. 10.1192/bjp.bp.106.033670 17906243

[32] Demjaha A Egerton A Murray RM Kapur S Howes OD Stone JM. Antipsychotic treatment resistance in schizophrenia associated with elevated glutamate levels but normal dopamine function. Biol Psychiatry (2014) 75:e11–13. 10.1016/j.biopsych.2013.06.011 23890739

[33] Viechtbauer W Conducting meta-analyses in R with the metafor package. J Stat Softw. (2010) 36:1–48. 10.18637/jss.v036.i03

[34] Brandt AS Unschuld PG Pradhan S Lim IAL Churchill G Harris AD Age-related changes in anterior cingulate cortex glutamate in schizophrenia: a (1)H MRS Study at 7Tesla. Schizophr Res. (2016) 172:101–5. 10.1016/j.schres.2016.02.017 26925800 PMC4821673

[35] Bustillo JR Chen H Jones T Lemke N Abbott C Qualls C. Increased glutamine in patients undergoing long-term treatment for schizophrenia: a proton magnetic resonance spectroscopy study at 3 T. JAMA Psychiatry (2014) 71:265–72. 10.1001/jamapsychiatry.2013.3939 24402128 PMC8185982

[36] Chiappelli J Rowland LM Wijtenburg SA Muellerklein F Tagamets M McMahon RP. Evaluation of myo-inositol as a potential biomarker for depression in schizophrenia. Neuropsychopharmacology (2015) 40:2157–64. 10.1038/npp.2015.57 25722115 PMC4613604

[37] Chiu PW Lui SSY Hung KSY Chan RCK Chan Q Sham PC. *In vivo* gamma-aminobutyric acid and glutamate levels in people with first-episode schizophrenia: a proton magnetic resonance spectroscopy study. Schizophr Res. (2017) 193:295–303. 10.1016/j.schres.2017.07.021 28751130

[38] Delamillieure P Constans J-M Fernandez J Brazo P Benali K Courthéoux P. Proton magnetic resonance spectroscopy (1H MRS) in schizophrenia: investigation of the right and left hippocampus, thalamus, and prefrontal cortex. Schizophr Bull. (2002) 28:329–39. 10.1093/oxfordjournals.schbul.a006942 12693438

[39] Lutkenhoff ES van Erp TG Thomas MA Therman S Manninen M Huttunen MO. Proton MRS in twin pairs discordant for schizophrenia. Mol Psychiatry (2010) 15:308–18. 10.1038/mp.2008.87 18645571

[40] Ongür D Prescot AP McCarthy J Cohen BM Renshaw PF. Elevated gamma-aminobutyric acid levels in chronic schizophrenia. Biol Psychiatry (2010) 68:667–70. 10.1016/j.biopsych.2010.05.016 20598290 PMC2942977

[41] Ongür D Jensen JE Prescot AP Stork C Lundy M Cohen BM. Abnormal glutamatergic neurotransmission and neuronal-glial interactions in acute mania. Biol Psychiatry (2008) 64:718–26. 10.1016/j.biopsych.2008.05.014 18602089 PMC2577764

[42] Reid MA Stoeckel LE White DM Avsar KB Bolding MS Akella NS. Assessments of function and biochemistry of the anterior cingulate cortex in schizophrenia. Biol Psychiatry (2010) 68:625–33. 10.1016/j.biopsych.2010.04.013 20570244 PMC2953853

[43] Rowland LM Pradhan S Korenic S Wijtenburg SA Hong LE Edden RA. Elevated brain lactate in schizophrenia: a 7 T magnetic resonance spectroscopy study. Transl Psychiatry (2016) 6:e967. 10.1038/tp.2016.239 27898072 PMC5290358

[44] Shirayama Y Obata T Matsuzawa D Nonaka H Kanazawa Y Yoshitome E. Specific metabolites in the medial prefrontal cortex are associated with the neurocognitive deficits in schizophrenia: a preliminary study. Neuroimage (2010) 49:2783–90. 10.1016/j.neuroimage.2009.10.031 19850131

[45] Taylor R Osuch EA Schaefer B Rajakumar N Neufeld RWJ Théberge J. Neurometabolic abnormalities in schizophrenia and depression observed with magnetic resonance spectroscopy at 7 T. BJ Psych Open (2017) 3:6–11. 10.1192/bjpo.bp.116.003756 28243459 PMC5288640

[46] Tayoshi S Sumitani S Taniguchi K Shibuya-Tayoshi S Numata S Iga J. Metabolite changes and gender differences in schizophrenia using 3-Tesla proton magnetic resonance spectroscopy (1H-MRS). Schizophr Res. (2009) 108:69–77. 10.1016/j.schres.2008.11.014 19097753

[47] Theberge J Al-Semaan Y Williamson PC Menon RS Neufeld RWJ Rajakumar N. Glutamate and glutamine in the anterior cingulate and thalamus of medicated patients with chronic schizophrenia and healthy comparison subjects measured with 4.0-T proton MRS. Am J Psychiatry (2003) 160:2231–3. 10.1176/appi.ajp.160.12.2231 14638596

[48] Théberge J Bartha R Drost DJ Menon RS Malla A Takhar J. Glutamate and glutamine measured with 4.0 T proton MRS in never-treated patients with schizophrenia and healthy volunteers. Am J Psychiatry (2002) 159:1944–6. 10.1176/appi.ajp.159.11.1944 12411236

[49] Yasukawa R Miyaoka T Mizuno S Inagaki T Horiguchi J Oda K. Proton magnetic resonance spectroscopy of the anterior cingulate gyrus, insular cortex and thalamus in schizophrenia associated with idiopathic unconjugated hyperbilirubinemia (Gilbert's syndrome). J Psychiatry Neurosci. (2005) 30:416–22. 16327875 PMC1277024

[50] Shimon H Sobolev Y Davidson M Haroutunian V Belmaker RH Agam G. Inositol levels are decreased in postmortem brain of schizophrenic patients. Biol Psychiatry (1998) 44:428–32. 10.1016/S0006-3223(98)00071-7 9777173

[51] Benes FM Davidson J Bird ED. Quantitative cytoarchitectural studies of the cerebral cortex of schizophrenics. Arch Gen Psychiatry (1986) 43:31–5. 10.1001/archpsyc.1986.01800010033004 3942472

[52] Cotter D Mackay D Chana G Beasley C Landau S Everall IP. Reduced neuronal size and glial cell density in area 9 of the dorsolateral prefrontal cortex in subjects with major depressive disorder. Cereb Cortex (2002) 12:386–94. 10.1093/cercor/12.4.386 11884354

[53] Rajkowska G Miguel-Hidalgo JJ Makkos Z Meltzer H Overholser J Stockmeier C. Layer-specific reductions in GFAP-reactive astroglia in the dorsolateral prefrontal cortex in schizophrenia. Schizophr Res. (2002) 57:127–38. 10.1016/S0920-9964(02)00339-0 12223243

[54] Cotter D Mackay D Landau S Kerwin R Everall I. Reduced glial cell density and neuronal size in the anterior cingulate cortex in major depressive disorder. Arch Gen Psychiatry (2001) 58:545–53. 10.1001/archpsyc.58.6.545 11386983

[55] Öngür D Drevets WC Price JL. Glial reduction in the subgenual prefrontal cortex in mood disorders. Proc Natl Acad Sci USA. (1998) 95:13290–5. 9789081 10.1073/pnas.95.22.13290 PMC23786

[56] Selemon LD Rajkowska G Goldman-Rakic PS. Abnormally high neuronal density in the schizophrenic cortex: a morphometric analysis of prefrontal area 9 and occipital area 17. Arch Gen Psychiatry (1995) 52:805–18. 10.1001/archpsyc.1995.03950220015005 7575100

[57] Harrison PJ. Postmortem studies in schizophrenia. Dialog Clin Neurosci. (2000) 2:349–57. 22033474 10.31887/DCNS.2000.2.4/pharrison PMC3181616

[58] Schneider M Koch M. Behavioral and morphological alterations following neonatal excitotoxic lesions of the medial prefrontal cortex in rats. Exp Neurol. (2005) 195:185–98. 10.1016/j.expneurol.2005.04.014 15935347

[59] Merritt K Egerton A Kempton MJ Taylor MJ McGuire PK. Nature of glutamate alterations in schizophrenia: a meta-analysis of proton magnetic resonance spectroscopy studies. JAMA Psychiatry (2016) 73:665–74. 10.1001/jamapsychiatry.2016.0442 27304221

[60] Coupland NJ Ogilvie CJ Hegadoren KM Seres P Hanstock CC Allen PS. Decreased prefrontal myo-inositol in major depressive disorder. Biol Psychiatry (2005) 57:1526–34. 10.1016/j.biopsych.2005.02.027 15953489

[61] Smiałowska M Szewczyk B Wozniak M Wawrzak-Wleciał A Domin H. Glial degeneration as a model of depression. Pharmacol Rep. (2013) 65:1572–9. 10.1016/S1734-1140(13)71518-4 24553005

[62] Gregory A Mallikarjun P Upthegrove R. Treatment of depression in schizophrenia: systematic review and meta-analysis. Br J Psychiatry (2017) 211:198–204. 10.1192/bjp.bp.116.190520 28882827

[63] McGlashan TH Bardenstein KK. Gender differences in affective, schizoaffective, and schizophrenic disorders. Schizophr Bull. (1990) 16:319–29. 2374887 10.1093/schbul/16.2.319

[64] Seelan RS Pisano MM Greene RM Casanova MF Parthasarathy RN. Differential methylation of the gene encoding myo-inositol 3-phosphate synthase (Isyna1) in rat tissues. Epigenomics (2011) 3:111–24. 10.2217/epi.10.73 21841945 PMC3154894

[65] McCarthy MM. Sex differences in neuroimmunity as an inherent risk factor. Neuropsychopharmacology (2018). 10.1038/s41386-018-0138-1. [Epub ahead of print]. 29977075 PMC6235925

[66] Santos-Galindo M Acaz-Fonseca E Bellini MJ Garcia-Segura LM. Sex differences in the inflammatory response of primary astrocytes to lipopolysaccharide. Biol Sex Differ. (2011) 2:7. 10.1186/2042-6410-2-7 21745355 PMC3143074

[67] Acaz-Fonseca E Avila-Rodriguez M Garcia-Segura LM Barreto GE. Regulation of astroglia by gonadal steroid hormones under physiological and pathological conditions. Prog Neurobiol. (2016) 144:5–26. 10.1016/j.pneurobio.2016.06.002 27283249

[68] Marsman A van den Heuvel MP Klomp DWJ Kahn RS Luijten PR Hulshoff Pol HE Glutamate in schizophrenia: a focused review and meta-analysis of 1H-MRS Studies. Schizophr Bull. (2011) 39:120–9. 10.1093/schbul/sbr069 21746807 PMC3523901

[69] Kraguljac NV Reid M White D Jones R den Hollander J Lowman D. Neurometabolites in schizophrenia and bipolar disorder – a systematic review and meta-analysis. Psychiatry Res. (2012) 203:111–25. 10.1016/j.pscychresns.2012.02.003 22981426 PMC3466386

[70] Glausier JR Lewis DA. Dendritic spine pathology in schizophrenia. Neuroscience (2013) 251:90–107. 10.1016/j.neuroscience.2012.04.044 22546337 PMC3413758

[71] Dogan AE Yuksel C Du F Chouinard V-A Öngür D. Brain lactate and pH in schizophrenia and bipolar disorder: a systematic review of findings from magnetic resonance studies. Neuropsychopharmacology (2018) 43:1681–90. 10.1038/s41386-018-0041-9 29581538 PMC6006165

[72] Shi D Xu S Waddell J Scafidi S Roys S Gullapalli RP. Longitudinal *in vivo* developmental changes of metabolites in the hippocampus of Fmr1 knockout mice. J Neurochem. (2012) 123:971–81. 10.1111/jnc.12048 23046047 PMC3957333

[73] Plitman E Fuente-Sandoval C de la Reyes-Madrigal F Chavez S Gómez-Cruz G León-Ortiz P. Elevated myo-inositol, choline, and glutamate levels in the associative striatum of antipsychotic-naive patients with first-episode psychosis: a proton magnetic resonance spectroscopy study with implications for glial dysfunction. Schizophr Bull. (2015) 42:415–24. 10.1093/schbul/sbv118 26320195 PMC4753594

[74] Auer DP Wilke M Grabner A Heidenreich JO Bronisch T Wetter TC. Reduced NAA in the thalamus and altered membrane and glial metabolism in schizophrenic patients detected by 1H-MRS and tissue segmentation. Schizophr Res. (2001) 52:87–99. 10.1016/S0920-9964(01)00155-4 11595395

[75] Chang L Friedman J Ernst T Zhong K Tsopelas ND Davis K. Brain metabolite abnormalities in the white matter of elderly schizophrenic subjects: implication for glial dysfunction. Biol Psychiatry (2007) 62:1396–404. 10.1016/j.biopsych.2007.05.025 17693392 PMC2222890

[76] Harwood AJ. Lithium and bipolar mood disorder: the inositol-depletion hypothesis revisited. Mol Psychiatry (2005) 10:117–26. 10.1038/sj.mp.4001618 15558078

[77] Konopaske GT Dorph-Petersen K-A Sweet RA Pierri JN Zhang W Sampson AR. Effect of chronic antipsychotic exposure on astrocyte and oligodendrocyte numbers in macaque monkeys. Biol Psychiatry (2008) 63:759–65. 10.1016/j.biopsych.2007.08.018 17945195 PMC2386415

[78] Szulc A Galinska B Tarasow E Dzienis W Kubas B Konarzewska B. The effect of risperidone on metabolite measures in the frontal lobe, temporal lobe, and thalamus in schizophrenic patients. a proton magnetic resonance spectroscopy (1H MRS). Pharmacopsychiatry (2005) 38:214–9. 10.1055/s-2005-873156 16189748

[79] Bracken BK Rouse ED Renshaw PF Olson DP. T2 relaxation effects on apparent N-acetylaspartate concentration in proton magnetic resonance studies of schizophrenia. Psychiatry Res. (2013) 213:142–53. 10.1016/j.pscychresns.2013.03.005 23769421 PMC3748739

[80] Ongür D Prescot AP Jensen JE Rouse ED Cohen BM Renshaw PF. T2 relaxation time abnormalities in bipolar disorder and schizophrenia. Magn Reson Med. (2010) 63:1–8. 10.1002/mrm.22148 19918902

[81] Williamson P Pelz D Merskey H Morrison S Karlik S Drost D. Frontal, temporal, and striatal proton relaxation times in schizophrenic patients and normal comparison subjects. Am J Psychiatry (1992) 149:549–51. 10.1176/ajp.149.4.549 1554045

[82] Li Q Leung YO Zhou I Ho LC Kong W Basil P. Dietary supplementation with n-3 fatty acids from weaning limits brain biochemistry and behavioural changes elicited by prenatal exposure to maternal inflammation in the mouse model. Transl Psychiatry (2015) 5:e641. 10.1038/tp.2015.126 26393487 PMC5068805

[83] McNamara RK Jandacek R Tso P Weber W Chu W-J Strakowski SM. Low docosahexaenoic acid status is associated with reduced indices in cortical integrity in the anterior cingulate of healthy male children: a 1H MRS Study. Nutr Neurosci. (2013) 16:183–90. 10.1179/1476830512Y.0000000045 23582513 PMC4101902

[84] Mukai T Kishi T Matsuda Y Iwata N. A meta-analysis of inositol for depression and anxiety disorders. Hum Psychopharmacol. (2014) 29:55–63. 10.1002/hup.2369 24424706

[85] Levine J Barak Y Gonzalves M Szor H Elizur A Kofman O. Double-blind, controlled trial of inositol treatment of depression. Am J Psychiatry (1995) 152:792–4. 10.1176/ajp.152.5.792 7726322

[86] Levine J Umansky R Ezrielev G Belmaker RH. Lack of effect of inositol treatment in chronic schizophrenia. Biol Psychiatry (1993) 33:673–5. 10.1016/0006-3223(93)90112-Q 8329500

[87] Firth J Stubbs B Sarris J Rosenbaum S Teasdale S Berk M. The effects of vitamin and mineral supplementation on symptoms of schizophrenia: a systematic review and meta-analysis. Psychol Med. (2017) 47:1515–27. 10.1017/S0033291717000022 28202095

[88] Elberling TV Danielsen ER Rasmussen AK Feldt-Rasmussen U Waldemar G Thomsen C. Reduced myo-inositol and total choline measured with cerebral MRS in acute thyrotoxic Graves' disease. Neurology (2003) 60:142–5. 10.1212/01.WNL.0000038911.07643.BF 12525741

[89] Haris M Cai K Singh A Hariharan H Reddy R. *In vivo* mapping of brain myo-inositol. Neuroimage (2011) 54:2079–85. 10.1016/j.neuroimage.2010.10.017 20951217 PMC3013615

[90] Howes OD McCutcheon R. Inflammation and the neural diathesis-stress hypothesis of schizophrenia: a reconceptualization. Transl Psychiatry (2017) 7:e1024. 10.1038/tp.2016.278 28170004 PMC5438023

[91] Shawcross DL Balata S Olde Damink SWM Hayes PC Wardlaw J Marshall I. Low myo-inositol and high glutamine levels in brain are associated with neuropsychological deterioration after induced hyperammonemia. Am J Physiol Gastrointest Liver Physiol. (2004) 287:G503–9. 10.1152/ajpgi.00104.2004 15130875

[92] Parker CA Nabulsi N Holden D Lin S Cass T Labaree D. Evaluation of 11C-BU99008, a PET ligand for the imidazoline2 binding sites in rhesus brain. J Nucl Med. (2014) 55:838–44. 10.2967/jnumed.113.131854 24711648

[93] Reid MA Salibi N White DM Gawne TJ Denney TS Lahti AC. 7T Proton magnetic resonance spectroscopy of the anterior cingulate cortex in first-episode schizophrenia. Schizophr Bull. (2018). 10.1093/schbul/sbx190. [Epub ahead of print]. 29385594 PMC6293230

